# The state of adolescent menstrual health in low- and middle-income countries and suggestions for future action and research

**DOI:** 10.1186/s12978-021-01082-2

**Published:** 2021-02-08

**Authors:** Marina Plesons, Archana Patkar, Jenelle Babb, Asanthi Balapitiya, Flo Carson, Bethany A. Caruso, Margarita Franco, Maja Manzenski Hansen, Jacquelyn Haver, Andisheh Jahangir, Caroline W. Kabiru, Ephraim Kisangala, Penelope Phillips-Howard, Aditi Sharma, Marni Sommer, Venkatraman Chandra-Mouli

**Affiliations:** 1 UNDP-UNFPA-UNICEF-WHO-World Bank Special Programme of Research, Development and Research Training in Human Reproduction (HRP), Department of Sexual and Reproductive Health and Research, World Health Organization, Geneva, Switzerland; 2 Gender, Human Rights and Community Engagement Department, UNAIDS, Geneva, Switzerland; 3 Regional Bureau for Education for the Asia-Pacific, UNESCO, Bangkok, Thailand; 4 Health Promotion Bureau, Ministry of Health, Colombo, Sri Lanka; 5 Foreign, Commonwealth and Development Office, London, UK; 6 Hubert Department of Global Health, Rollins School of Public Health, Emory University, Atlanta, USA; 7 Save the Children El Salvador, San Salvador, El Salvador; 8 United Nations Population Fund, Dar es Salaam, Tanzania; 9 Save the Children USA, Washington, DC USA; 10 WoMena Knowledge Management Team, WoMena Denmark, Copenhagen, Denmark; 11 African Population and Health Research Center, Nairobi, Kenya; 12 African Centre for Systematic Review and Knowledge Translation, College of Health Sciences, Makerere University, Kampala, Uganda; 13 Department of Clinical Sciences, Liverpool School of Tropical Medicine, Liverpool, UK; 14 Department of Public Health Sciences, College of Medicine, Pennsylvania State University, State College, USA; 15 Department of Sociomedical Sciences, Mailman School of Public Health, New York, USA

**Keywords:** Menstruation, Menstrual health, Menstrual hygiene, Menstrual hygiene management, Adolescent health, Adolescent sexual and reproductive health

## Abstract

In recognition of the opportunity created by the increasing attention to menstrual health at global, regional, and national levels, the World Health Organization’s Department of Sexual and Reproductive Health and Research and the UNDP-UNFPA-UNICEF-WHO-World Bank Special Programme of Research, Development and Research Training in Human Reproduction convened a global research collaborative meeting on menstrual health in adolescents in August 2018. Experts considered nine domains of menstrual health (awareness and understanding; stigma, norms, and socio-cultural practices; menstrual products; water and sanitation; disposal; empathy and support; clinical care; integration with other programmes; and financing) and answered the following five questions: (1) What is the current situation? (2) What are the factors contributing to this situation? (3) What should the status of this domain of adolescent menstrual health be in 10 years? (4) What actions are needed to achieve these goals? (5) What research is needed to achieve these goals? This commentary summarizes the consensus reached in relation to these questions during the expert consultation. In doing so, it describes the state of adolescent menstrual health in low- and middle-income countries and sets out suggestions for action and research that could contribute to meeting the holistic menstrual health needs of adolescent girls and others who menstruate worldwide.

## Background

A growing body of evidence on menarche, menstruation, menstrual hygiene, and menstrual health^1^ among adolescent^2^ girls in low- and middle-income countries (LMICs) has revealed a somber, yet sadly unsurprising situation: menstruation continues to be shrouded in silence and stigma and remains a neglected issue in many places around the world [1]. Many girls are uninformed and unprepared for menstruation, and experience fear and anxiety upon reaching menarche [2, 3]. Mothers, other female relatives, and female peers are their main sources of information, but this information is often neither adequate nor timely [2]. Girls experience a variety of symptoms, such as pain, headaches, and fatigue, and—when combined with social and cultural taboos—they often cannot participate in household, educational, employment, social and/or religious activities [2, 3]. Few girls seek health care when they experience menstrual problems, but many use household remedies [2, 3]. Lastly, girls in rural and poor urban communities and humanitarian crisis settings are less likely to be able to access and/or use menstrual products to manage menstruation, and often lack access to soap, safe water, and functional and secure toilets/latrines with mechanisms for private disposal of used menstrual products [2–7]. These challenges have immediate and longer-term consequences for girls in relation to their confidence and self-efficacy, their ability to participate in day-to-day activities such as education and employment, and their health and wellbeing.

However, thanks to sustained and innovative advocacy by a range of stakeholders and a number of platforms, coalitions, and networks at global, regional, and national levels, menstrual health is increasingly recognized as a public health issue that is closely linked to several human rights and the attainment of the Sustainable Development Goals (SDGs) [8, 9]. As a result, governmental ministries/departments (health; education; gender; water, sanitation and hygiene (WASH), etc.), non-governmental organizations (NGOs), the private sector, and funders are increasingly ready and willing to act [1]. These positive intentions, though, are bound by the information and evidence currently available. Research thus far has predominately focused on the reasons for and consequences of poor menstrual hygiene, and action has largely focused on addressing girls’ menstruation-related needs in school settings and humanitarian crisis settings, and on improving girls’ access to menstrual products in wider contexts [10–12]. More recently, research has expanded to cover other issues, such as the factors driving the stigma surrounding menstruation and the needs of specific groups of girls, such as those with disabilities or chronic health conditions [13, 14]. There remains a lack of comparable data on menstruation across settings, at different points in the life-course, and among people with diverse gender identities, as well as a lack of evidence regarding the effectiveness, cost, and cost-effectiveness of interventions across relevant sectors [9]. Further, there is a lack of evidence on what works to promote and sustain integration, multi-sectoral coordination, and financing on menstrual health.

In recognition of the opportunity created by the increasing attention to menstrual health, the World Health Organization’s (WHO) Department of Sexual and Reproductive Health and Research and the UNDP-UNFPA-UNICEF-WHO-World Bank Special Programme of Research, Development and Research Training in Human Reproduction convened a global research collaborative meeting on menstrual health in adolescents in August 2018. The meeting brought together a diverse range of experts—including representatives from government, international organizations, NGOs, academia, and funders, along with young people themselves—and sought to map the state of the field on adolescent menstrual health and generate suggestions for future action and for research. Specifically, the experts considered nine domains of menstrual health (awareness and understanding; stigma, norms, and socio-cultural practices; menstrual products; water and sanitation; disposal; empathy and support; clinical care; integration with other programmes; and financing) and sought to reach consensus regarding the following five questions:

- What is the current situation?
- What are the factors contributing to this situation?
- What should the status of this domain of adolescent menstrual health be in 10 years?
- What actions are needed to achieve these goals?
- What research is needed to achieve these goals?

The sections that follow summarize the consensus reached in relation to these questions during the expert consultation, by domain. In doing so, this commentary describes the state of adolescent menstrual health in LMICs and sets out suggestions for action and research that could contribute to meeting the holistic menstrual health needs of adolescent girls and others who menstruate worldwide.

It is important to note that while these domains of menstrual health are addressed separately in this commentary, there is also a need to better understand how these domains, including their determinants and consequences, reinforce each other. Additionally, while this commentary discusses the actions and research needed to improve menstrual health for adolescent girls and others who menstruate generally, specific attention is required to ensure that action and research include and benefit those who are socially and economically marginalized. Finally, it is worth noting that this commentary is not a systematic review, that it did not seek to describe similarities and differences in viewpoints of various stakeholder groups on the issues discussed, and that the status of adolescent menstrual health and the responses to it have continued to evolve in the time since this expert consultation.

## Awareness and understanding

### What is the current situation?

Children, adolescents, and adults around the world lack awareness, knowledge, and understanding of menstrual health. The knowledge they do have is often tinged with misconceptions. This is seen as a key factor that contributes to many challenges related to menstrual health [2, 3, 15].

### What are the factors contributing to this situation?

Firstly, there is refusal, reluctance, discomfort, and/or lack of interest across populations to talk about menstruation because it is associated with sexuality and reproduction, and because it is seen as a girls and women’s issue to be dealt with by girls, women, and others who menstruate themselves [16]. Secondly, the primary sources of information and advice related to menstruation for adolescent girls and others who menstruate in most LMICs are mothers and other female family members. However, these individuals (and others who may communicate about menstruation) themselves have important knowledge gaps and misconceptions, and often pass on these knowledge gaps and misconceptions inadvertently [17]. Thirdly, most health education programmes in school and community settings do not adequately address menstruation, or do not address it at all [18]. When menstruation is addressed, the information provided typically focuses on the biology of menstruation and lacks information on its association with sexual and reproductive health (SRH), as well as practical guidance for managing menstruation, including pain, and opportunities for care and support if needed [19, 20]. Additionally, such information is often directed only to girls and women and not to boys and men, which is required for interventions to be truly gender transformative [21].

### What should the status of this domain of adolescent menstrual health be in 10 years?

All people should see menstruation as a healthy and normal biological phenomenon and should be aware of and knowledgeable about menstruation, how and why it occurs, and how to manage it (inclusive of flow, pain, and other key issues) safely and with dignity. Children, adolescents, and adults should be provided with timely, accurate, and up-to-date information and education on menstrual health, including through comprehensive sexuality education that is developmentally appropriate and culturally sensitive. Menstruation should be recognized as an important health and education issue that needs to be understood and addressed in an empathetic and meaningful way by all people—those who menstruate and those who do not—in homes, schools, workplaces, communities, and by policy-makers and the media. It should also be recognized as a useful and potentially powerful entry point for addressing SRH and rights more broadly.

### What actions are needed to achieve these goals?

Where supportive and evidence-based policies do not yet exist, policy-makers must be made aware of and convinced of the value of establishing policies on the provision of information and education on menstrual health to children, adolescents, and adults, such as those that stipulate the provision of comprehensive sexuality education. Once formulated, such policies must be translated into adequately funded strategies/plans, which must then be implemented, monitored and evaluated with quality and equity considerations through a combination of channels, such as schools, radio, television, print and social media, appropriate to the local context.

### What research is needed to achieve these goals?

Firstly, consensus must be reached on a standard set of validated indicators to assess individuals’ awareness, knowledge, and understanding on specific areas of menstruation, in order to identify needs, support advocacy, inform policies and programmes, target resources, and enable comparison within and across countries and over time [22]. Secondly, evidence is required on the effectiveness, cost, and cost-effectiveness of programmatic interventions to deliver developmentally appropriate information and education on menstruation, and on how they can be implemented at scale, with quality and equity.

## Stigma, norms, and socio-cultural practices

### What is the current situation?

Menstruation is perceived by many people as dirty, messy, polluting, and impure, and there is a sense that it is shameful and should be concealed. This, in turn, leads to girls and others who menstruate being and feeling isolated and/or expected to deal with menstruation on their own to avoid what is perceived as unpleasant, or as physical and spiritual contamination [23].

While the intensity of experiences and perceptions related to menstrual stigma vary by context, such stigma is global, longstanding, and deep-seated [24, 25]. This stigma—internalized by girls and others who menstruate or expressed by others—as well as discriminatory practices that relate to it, have negative impacts and important consequences for the well-being of girls, women, and others who menstruate, including their agency, mobility, dignity, and health. In some places, more extreme forms of discrimination imposed on girls and others who menstruate, such as the harmful practice of Chhaupadi^3^ that was widely practiced in parts of Nepal [26].

### What are the factors contributing to this situation?

Firstly, these perceptions are fueled by harmful gender norms, gender inequality, and related power structures in households and institutions, as well as by a lack of awareness, knowledge, and understanding about menstruation [6, 13, 27]. Secondly, they are exacerbated by the lack of access to safe, private, and affordable water, sanitation, and disposal systems and menstrual products and materials, which hinders girls and others who menstruate’s abilities to manage menstruation [12, 28]. Thirdly, they are reinforced by the lack of attention to menstrual health in health and education programmes (e.g. comprehensive sexuality education), and by negative portrayals in the media. Lastly, they are reproduced by families and communities, for example through rites of passage [8].

### What should the status of this domain of adolescent menstrual health be in 10 years?

Stigma and harmful norms and socio-cultural practices surrounding menstruation should be dismantled to improve girls, women, and others who menstruate’s experiences of menstruation and to enable gender equality [13]. This means that all children, adolescents, and adults should view menstruation as healthy and normal. Menstruation should be seen as an important subject that is addressed by families, schools, communities, governments, and media without shame and embarrassment. It needs to be portrayed in a accurate, empathetic, and meaningful way that encompasses its social and biological aspects. Girls and others who menstruate should have access to the safe, private, and affordable water, sanitation, and disposal systems and menstrual products and materials they need to manage their menstrual periods effectively, in the context of a supportive environment, thereby ending the perception that menstruation is dirty and messy.

### What actions are needed to achieve these goals?

Firstly, evidence-based, culturally appropriate, and gender transformative policies and programmes must be put in place to directly address stigma and normalize menstruation, situated within wider efforts to address harmful gender norms and gender inequality [13]. Secondly, these policies and programmes should:

(i) Deliver information and education to children, adolescents, and adults about menstruation, through a combination of channels including comprehensive sexuality education appropriate to the local context. This information and education should facilitate a shared understanding and acceptance of menstruation as a natural biological phenomenon.
(ii) Engage children, adolescents, and adults in gender-transformative social norm and behavior change interventions.
(iii) Ensure that girls and others who menstruate have access to the safe, private, and affordable water, sanitation, and disposal systems and menstrual products and materials they need to manage their periods safely and with dignity.

Thirdly, these policies must be translated into adequately funded strategies/plans, which must then be implemented, monitored, and evaluated with quality and equity considerations. Lastly, media must report on menstruation in a biologically accurate, empathetic, and meaningful manner.

### What research is needed to achieve these goals?

Firstly, consensus must be reached on a standard set of validated indicators to assess stigma, gender norms, and gender inequality related to menstruation. Secondly, evidence is required on the effectiveness, cost, and cost-effectiveness of interventions to address stigma, as part of broader interventions to address harmful gender norms and gender inequality, and on how they can be implemented at scale, with quality and equity.

## Menstrual products

### What is the current situation?

There are a number of menstrual products and materials available for use across the globe, with varying degrees of quality, accessibility, and acceptability. Each has advantages and disadvantages (Table 1). Amongst all commercial products, single-use products appear to be the most widely available. In many places, they also appear to be the most widely used because they are the products that people are most aware of, that people find most convenient and acceptable, that are most widely available in commercial outlets, and that are provided free of charge by government and NGO programmes. Among those in poorer, less educated, and more rural communities, cloth and/or home-made materials (e.g. cloth packaged with cotton) and/or make-shift materials (e.g. paper towels and toilet tissue), appear to be used more frequently than in higher-income environments [15, 29].

**Table 1:** Advantages and disadvantages of common menstrual products/materials

Type of product/material	Advantages	Disadvantages
Pads: single-use	Often preferred by users/programmes; numerous brands; absorbent; convenient; accessible	Repeat expense; variable quality; require disposal mechanism; include plastic/pollutes; require users to wear pants/underwear
Pads: Reusable [30, 31]	Reusable; absorbent; cheap; accessible; relatively environment-friendly	Require water, soap, and a private space and sunlight to dry; variable quality; require users to wear pants/underwear; debate about risk of infection
Tampons [15]	Absorbent; numerous brands; does not require users to wear pants/underwear; permits users to participate in all activities	Repeat/higher expense; may violate norms in some contexts around vaginal insertion; require disposal mechanism; include plastic/pollutes; variable accessibility; require training/support on safe use; debate about risk of infection
Cups [32–34]	Absorbent; does not require user to wear pants/underwear; numerous brands; permits users to participate in all activities, environment-friendly	Initial/upfront expense; may violate norms in some contexts around vaginal insertion; include plastic/pollutes; variable accessibility; require training/support on safe use
Cloth [15, 35]	Reusable; cheap; accessible; environment-friendly	Require water, soap, a private space and sunlight to dry; debate about risk of infection; require users to wear pants/underwear
Sea sponges	Absorbent; reusable; natural; does not require users to wear pants/underwear; permits users to participate in all activities; environment-friendly	Limited availability; can contain bacteria/sand/sharp shells/hard coral; require training on safe use
Period panties	Reusable; absorbent; permits users to participate in all activities	Initial/upfront expense; variable accessibility; require water, soap, a private space and sunlight to dry; may require use with tampons or pads for heavy periods

However, many girls, women, and others who menstruate lack access to menstrual products and materials that meet their needs and preferences, or the clothing (e.g. underwear) needed to use them. This hampers their agency, their health and well-being—both psychological and physical—and their ability to participate in daily activities, such as education or employment.

### What are the factors contributing to this situation?

The range of products and materials available globally has grown and continues to grow because of successful advocacy, proliferation of production (by corporations and social entrepreneurs, and by health and social development projects/programmes), and the heavy focus on products in menstrual health and/or hygiene-related policies and programmes [36].

Some governments have made efforts to remove/reduce taxes on products or to provide free products to all or some girls, women, and others who menstruate. These efforts have been ad hoc, inconsistent, have not always reached those for whom they were intended, and have often not been sufficiently monitored and evaluated to examine their impact and their sustainability. The lack of access to menstrual products and materials that meet the needs and preferences of girls, women, and others who menstruate persists due to factors ranging from poverty and gendered power imbalances in control over household resources, to distribution.

### What should the status of this domain of adolescent menstrual health be in 10 years?

Girls and others who menstruate should be aware of and have access to a range of affordable products and materials that meet their personal needs and preferences and that are safe and efficacious, in line with national and global standards. They should also have access to soap and safe and private water, sanitation, and disposal systems that are needed to use these products/materials [37]. Products and materials should not be taxed, should be available at a reasonable price for those who are able to pay, and should be subsidized/free-of-charge for those who are unable to pay.

In line with these goals, governments should work with manufacturers to institute standards across product manufacturing and supply chains that ensure human and environmental health and safety. Product manufacturers should be obliged to meet set standards and should be encouraged to seek out innovative approaches to produce more environmentally sustainable products. Marketeers should be obliged to sell regulatory-approved products at the recommended prices, and should be held accountable for doing so. And governments and NGOs that promote/distribute products should be obliged to use government-certified products.

### What actions are needed to achieve these goals?

Firstly, standards for efficacy and for health and environmental safety of the full range of menstrual products and materials must be developed. Manufacturers should be made aware of these standards, and they should be enforced by relevant national/international authorities with accountability structures in place to address non-compliance. International monitoring systems should be established to ensure safety and effectiveness of products, and to enable comparison between products.

Secondly, evidence-based policies that require the sale/distribution of a range of subsidized and/or free-of-charge products must be established, and governments must be held accountable for applying and monitoring them. As part of these efforts, governments will need to withdraw taxes that increase the price of these essential products and set recommended prices for their sale. These policies must be translated into strategies/plans, which must then be implemented, monitored, and evaluated with quality and equity considerations.

Lastly, communication programmes—through mainstream media and health promotion, as well as through formal and non-formal comprehensive sexuality education—need to be put in place to inform girls and others who menstruate, as well as their families and communities, about the full range of products and materials available (including cloth and/or home-made materials) that might meet their personal needs and preferences, as well as how to use these products/materials safely and efficaciously. These efforts must be combined with initiatives to inform girls and others who menstruate, as well as their families and communities, about the importance of safe disposal of menstrual products/materials.

### What research needs to be undertaken to achieve these goals?

Evidence is required on the health, efficacy, and environmental safety of the full range of menstrual products and materials; on the availability and cost of menstrual products/materials (where applicable); and on the levels of use by different groups, including their ability to use the products/materials safely and the factors hindering and helping their use of these products/materials.

## Water and sanitation

### What is the current situation?

Access to safe water and sanitation improved worldwide during the Millennium Development Goal era and has continued to improve during the SDG era. However, access is still uneven and inequitable in homes, schools, workplaces, health care facilities, and in public places within communities [38–41]. This severely hampers the ability of girls, women, and others who menstruate, especially those who are socially and economically marginalized, to manage menstruation safely and with dignity [42]. Limited access to safe water and sanitation can hinder girls and others who menstruate from being able to move about freely for educational, professional, recreational, and/or social reasons during their menstrual periods, and can contribute to feelings of inadequacy and shame [42, 43]. Finally, in some places, girls and others who menstruate may be more vulnerable to sexual harassment and violence when public water sources and toilets/latrines lack privacy or are not near their homes and/or workplaces [43].

### What are the factors contributing to this situation?

There is still a lack of adequate investment in ensuring the availability of safe, private, and affordable water and sanitation systems, and in ensuring equitable access to such infrastructure and services [44]. While guidelines and standards have been developed to ensure gender is accounted for in the WASH sector, there is still limited acknowledgement of the needs and preferences of girls, women, and others who menstruate, and of their meaningful involvement in designing, executing, and assessing such efforts [45–48].

### What should the status of this domain of adolescent menstrual health be in 10 years?

All communities, including in homes, schools, workplaces, health care facilities, and public spaces, should have equitable—including gender equitable—access to clean, safe, private, physically accessible, and affordable water and sanitation systems.

### What actions are needed to achieve these goals?

Standards and guidance on expanding access to equitable—including gender equitable—clean, safe, private, physically accessible, and affordable water and sanitation systems need to be developed where they do not already exist. Additionally, evidence-based gender-equitable national policies to improve access to such infrastructure and services must be established. These policies must be translated into strategies/plans, which must then be implemented, monitored, and evaluated with quality and equity considerations.

### What research needs to be undertaken to achieve these goals?

Evidence is required on whose needs for safe, private, and affordable water and sanitation systems in relation to menstruation are met, and whose needs are not. Evidence is also required on cost and cost-effective ways of meeting the water and sanitation needs in relation to menstruation for all girls, women, and others who menstruate, especially those who are socially and/or economically marginalized.

## Disposal

### What is the current situation?

Disposal of used menstrual products/materials is often overlooked in policies and programmes, despite estimates that over 12 billion disposable menstrual products are used per year and that these disposed products create approximately 6.3% of sewage-related debris along rivers and beaches [48, 50, 51]. Many of the disposal practices employed—including use of pit latrines, toilets, garbage, incineration/open burning, burying, and open dumping into ponds and fields—are damaging, rudimentary, and unregulated, yet may also be the only option available [48, 49, 52]. Furthermore, the health and safety ramifications, both for the people handling the materials and for the environment, are largely unknown [53]. Lastly, single-use menstrual products are of particular concern because they are non-biodegradable and their incineration—if not conducted under specified conditions and using recommended procedures—can release harmful toxins [54–56].

Additionally, the disposal of menstrual products can be a source of embarrassment and shame, especially when girls, women, and others who menstruate lack access to adequate disposal mechanisms [49]. For example, girls report walking to open dumping areas while hiding their used menstrual products when schools do not have bins in their toilets, or adequate private onsite disposal systems [57].

### What are the factors contributing to this situation?

Firstly, manufacturers of menstrual products have greater incentive to ensure their products are convenient for consumers to use than to ensure they are environmentally safe to dispose. Secondly, there are few regulations to ensure transparency regarding product content, manufacturing processes, and possible harmful impact on humans and the environment [56, 58]. Thirdly, there has been lack of adequate attention to, and investment in, waste management, including but not limited to menstrual products/materials [59]. Fourthly, the health and safety consequences of menstrual product/material disposal are generally considered the responsibility of those in charge of waste management, not of those in charge of menstrual product manufacturing and/or procurement. Similarly, the long-term environmental consequences of menstrual product/material disposal are often not acknowledged and/or addressed by researchers, policy-makers, programme implementers, and consumers [54].

### What should the status of this domain of adolescent menstrual health be in 10 years?

Governments should ensure that all girls, women, and others who menstruate have access to safe and adequate disposal methods for menstrual products/materials, as part of functional waste management systems. All girls, women, and others who menstruate should be aware of and able to use such disposal mechanisms. Further, product manufacturers should be obliged to meet safe disposal standards.

### What actions are needed to achieve these goals?

Culturally appropriate and financially viable standards for the safe disposal of the full range of menstrual products and materials need to be developed. In line with this action, manufacturers and waste management officials must be made aware of these standards through briefing sessions and certifications and held accountable for complying with them [60]. Additionally, evidence-based policies that require the safe disposal of products and materials must be developed. These policies must be translated into adequately funded strategies/plans, which must then be implemented, monitored, and evaluated with quality and equity considerations. Lastly, effective communication programmes need to be put in place to inform girls, women, and others who menstruate, as well as their families and communities, about proper disposal methods, and encourage them to use them.

### What research needs to be undertaken to achieve these goals?

Evidence is needed on the health and environmental safety and the environmental lifecycle of the full range of menstrual products and materials [61]. Evidence is also needed on the cost and cost-effectiveness of safe disposal solutions for various contexts and at different levels of volume for different sizes of populations.

## Empathy and support

### What is the current situation?

Many girls and others who menstruate do not receive the empathy and support they need regarding menstrual health from the people in their lives—at home, in school, in workplaces, and in their communities [25, 62]. On the contrary, they are sometimes humiliated or shamed by others [63]. For example, girls report being teased about menstruation by their peers, both boys and girls, and even by teachers in school [64]. This hampers girls and others who menstruate’s well-being, including their agency and confidence.

### What are the factors contributing to this situation?

Menstrual heatlth is commonly seen as something that does not require empathy and support. Instead, it is seen as something that girls, women, and others who menstruate need to deal with on their own. Even when individuals acknowledge that empathy and support are needed on this issue and wish to provide it, they are often constrained by norms that prohibit public acknowledgement about menstruation, as well as their own discomfort in doing so. Likewise, they are often constrained in providing empathy and support by their own knowledge gaps and misconceptions.

### What should the status of this domain of adolescent menstrual health be in 10 years?

Families, including parents/caregivers, and communities should view menstruation as healthy and normal, and recognize that girls and others who menstruate need empathy and understanding about menstruation and that they may need/want support. Further, there should be recognition that responding to these needs can improve the health and well-being of girls and others who menstruate, including their agency and confidence. Additionally, children, adolescents, and adults should be obliged by this widespread recognition to respond to the girls and others who menstruate in their lives with empathy and support about menstruation.

### What actions are needed to achieve these goals?

Firstly, policies must be established to inform and educate children, adolescents, and adults about menstruation, to normalize it, and to promote empathy and support for girls and others who menstruate [13]. These policies must be translated into adequately funded strategies/plans, which must then be implemented, monitored, and evaluated with quality and equity considerations. Secondly, media must report on this subject in an informed and empathetic manner. These activities must be situated within concerted wider efforts to address gender inequality.

### What research needs to be undertaken to achieve these goals?

See the research described in the sections on awareness, knowledge, and understanding, and on stigma, norms, and socio-cultural practices.

## Clinical care

### What is the current situation?

In many places, girls and others who menstruate do not get prompt and effective clinical care when they experience menstrual health problems, such as premenstrual syndrome, painful menstrual periods, irregular menstrual periods, excessive bleeding, and delayed or early onset of menstrual periods [65, 66]. Of those who do receive care, most receive traditional or modern remedies from their family members or friends, rather than from a health worker [2]. Many others cope with such problems on their own and in silence. Lack of access to prompt and effective clinical care hampers girls and others who menstruate’s functioning and their sense of well-being, including their agency and confidence. It also results in potentially serious medical problems that manifest with menstrual symptoms potentially not being diagnosed and treated [67, 68]. Finally, inadequate care represents a missed opportunity to integrate menstrual health into the wider package of essential SRH services and to link efforts to improve menstrual health with initiatives on related issues, such as contraception.

### What are the factors contributing to this situation?

Firstly, many girls and others who menstruate lack knowledge and understanding about menstruation, including how to recognize menstrual health problems if and when they occur. As a result, they may not know whether to seek clinical care or where to do so when they need it [68]. Secondly, girls and others who menstruate are generally expected to deal with menstruation on their own. As a result, they may not feel comfortable or able to seek clinical care when they need it. Thirdly, families and communities generally do not believe in the need for clinical care for menstrual health problems. As a result, girls and others who menstruate may not be supported in accessing clinical care if they seek it [2]. Fourthly, despite the expansion of the scope of health problems included in countries’ packages of health services, preventive and curative services for menstrual health problems are typically still not explicitly addressed [69, 70]. As a result, health workers are not obliged, trained, or prepared to provide these services [71]. Lastly, health services are generally not set up to provide clinical care to older children (i.e., those older than 5 years) and young adolescents (i.e., those 10–14 years), or to people with diverse gender identities, and may not be accessible to these groups [72]. As a result, both health workers and health systems are often not prepared to respond to the needs of these age groups and individuals in an appropriate and responsive manner, especially in regards to sexual and reproductive matters, including menstrual health [73].

### What should the status of this domain of adolescent menstrual health be in 10 years?

Girls and others who menstruate should know when, where, and how to seek clinical care for menstrual health when they need it, should be able to access respectful and effective advice and care for menstrual health from competent and empathic health workers when needed, and should be supported by their families and communities in doing so. Clinical care for menstrual health should be accepted by all as legitimate and important and should be recognized as a useful entry point for addressing SRH and rights more broadly. Further support for addressing menstrual health problems should be available through school health services and community-based programmes.

### What actions are needed to achieve these goals?

Firstly, standards and guidance must be developed on the provision of promotive, preventive, and curative clinical care services for menstrual health. Advocacy must be conducted for the inclusion of promotive, preventive, and curative clinical care services for menstrual health (in line with such standards and guidance) as an integral part of packages of essential SRH services in policies. Secondly, these policies must be translated into strategies/plans, which must then be implemented, monitored, and evaluated with quality and equity considerations. Thirdly, advocacy is needed for health systems to train and support health workers to provide clinical care for menstrual health through pre- and in-service training and other health worker capacity building approaches, and support them and hold them accountable for doing so. Further, clinical care for menstrual health should be included in other service delivery channels, such as in school health services and community-based programmes.

### What research needs to be undertaken to achieve these goals?

Evidence needs to be strengthened on the provision of promotive, preventive, and curative clinical care services for menstrual health, as well as feasible and acceptable means of delivering such services. Evidence is also needed on the positive and negative economic impacts of investing in preventive, promotive, and curative clinical care services for menstrual health, to feed into advocacy efforts.

## Integration with other programmes

### What is the current situation?

Even though multiple sectors need to contribute to meeting the menstrual health needs of girls, women, and others who menstruate, multi-sectoral coordination and integration with other programmes is a major challenge [12]. Sectors (i.e., WASH, education, health, etc.) that should make contributions often fail to include it in their agendas [28, 74, 75]. When individual sectors do make contributions, they often do so inadequately (e.g., improvement of WASH without integration of a gender lens) or through piecemeal actions (e.g., provision of menstrual products as a standalone intervention) [76]. When multiple sectors do make contributions, they often fail to integrate these actions for complementary and coordinated programme planning and monitoring and evaluation [9]. The exception appears to be when leadership recognizes menstrual health as important for health and gender equality and pushes the agenda forward on a continuous basis [77, 78]. Lastly, when menstrual health is included in broader multisectoral strategies and plans (e.g., on adolescent health), the menstrual health content is often limited in the design stage and further limited in the execution stage.

### What are the factors contributing to this situation?

At global and national levels, menstrual health continues to lack prioritization and adequate investment [8, 79]. Because of this, menstrual health is often not addressed in a meaningful way in international agendas and funders’ priorities or in national policies, strategies, and budgets. Further, the roles and responsibilities of different sectors are not articulated, and different sectors are not mandated to carry out their respective contributions, let alone ensure that these contributions are integrated with those of other sectors [8]. Underlying these challenges is the reality that intersectoral coordination—regardless of the issue it is meant to address—is difficult and generally weak.

### What should the status of this domain of adolescent menstrual health be in 10 years?

The holistic menstruation-related needs of girls, women, and others who menstruate should be adequately supported through effective complementary and coordinated contributions of different sectors at global, regional, national, and sub-national levels.

### What actions are needed to achieve these goals?

At the global level, standards and practical guidance must be developed on the package of multi-sectoral actions needed to normalize menstruation and support the holistic menstrual health needs of girls and others who menstruate, as well as approaches and tools to monitor and measure integration.[80] At global, regional, national, and sub-national levels, a shared understanding and vision of menstrual health as important for health and gender equality and of how to address it must be established. The roles and responsibilities of different sectors must be delineated and clarified. Committed and skilled leadership to champion the issue must be identified and engaged. Finally, coordination mechanisms with the authority and resources to direct and operationalize integrated action must be established.

### What research needs to be undertaken to achieve these goals?

Evidence is required on approaches that can be used to achieve effective integration and multisectoral action for menstrual health, in crisis and non-crisis contexts, as well as evidence on the added value of it.

## Financing

### What is the current situation?

There is more financing for menstrual health than ever before, but the level of investment is still insufficient to address the issue adequately. It is unclear which funders and investors (i.e., governments, foundations, NGOs, and the private sector) are allocating money for this work, how much money is available, what the money is and is not available for, which groups the money is and is not available for, and whether the best use is being made of the available money—in terms of selecting effective interventions and approaches and delivering them efficiently [81].

### What are the factors contributing to this situation?

Firstly, there is greater attention to menstruation in the context of health, development and human rights, and in the media. This has led to stronger calls for investment and corresponding action [18]. Secondly, there is greater political appetite to tackle this issue in some countries and greater investment in this area from some funders and investors. There are also many projects, as well as growing numbers of national programmes, which aim to address menstrual health [82]. Thirdly, policies to eliminate product taxes are in place in many countries. Fourthly, policies and programmes to increase equitable access to information and products for marginalized groups are in place in a growing number of countries [83].

### What should the status of this domain of menstrual health be in 10 years?

There should be adequate international and national resources for addressing menstrual health across the relevant sectors, with particular attention to socially and/or economically marginalized girls, women, and others who menstruate, including those in fragile and humanitarian crisis contexts. Additionally, programmes should account for and leverage financing from different sources for phased scale-up and sustainability of interventions to support the menstruation-related needs of girls, women, and others who menstruate. These programmes should be monitored and evaluated to ensure that the best use is being made of the available money.

### What actions are needed to achieve these goals?

Firstly, advocacy is needed for greater investment in this area by funders and investors. This could result from greater allocation of available resources (e.g., in comprehensive sexuality education and in adolescent health care), and from new resources directed to this area. Secondly, greater attention must be given to costing and cost-effectiveness in the context of research and implementation. Thirdly, incentives for manufacturers of menstrual products and a climate of healthy competition to drive down product prices must be created. This must be combined with advocacy for development of policies on tax exemption and on the sale/distribution of subsidized and/or free-of-charge products.

### What research needs to be undertaken to achieve these goals?

Evidence is needed on the resources required to deliver interventions to meet the menstrual health needs of girls, women, and others who menstruate (including the costs of products/materials, and of safe, private, and affordable water, sanitation, and disposal systems themselves)—at scale, with quality and equity. Evidence must be strengthened on the social and economic costs of not meeting the menstrual health needs of girls, women, and others who menstruate. Lastly, evidence is needed on the cost and cost-effectiveness of different interventions and intervention delivery approaches.

## Conclusion

There is much to be done—both in terms of action and research—to meet the holistic menstrual health needs of girls and others who menstruate worldwide. However, the moment is ripe for progress. With five years left to achieve the SDG agenda, this is an opportune time to advance the menstrual health agenda as a catalyst to advance multiple goals while also progressing SRH and rights. More so than ever before, there is widespread recognition of the need to address adolescents’ health and well-being and commitment to doing so [1]. Finally, there are thriving grassroots menstrual equity movements around the world, pushing for menstruation to be considered alongside efforts to build  gender equity and social justice. While the call to action for researchers, policy makers, programme implementers, and funders is considerable, so is the opportunity currently at hand.

## Data Availability

Not applicable.

## Abbreviations

LMIC: Low- and middle-income country
NGO: Non-governmental organization
SDG: Sustainable development goal
SRH: Sexual and reproductive health
WASH: Water, sanitation, and hygiene
WHO: World Health Organization

## Footnotes

1 Hereafter, this group of topics is referred to as ‘menstrual health.’.

2 WHO defines adolescents as those between the ages of 10 and 19 years.

3 Chhaupadi is a harmful practice in parts of Nepal in which girls, women, and others who menstruate are required to stay outside of the house—often in a cow shed or similar structure—when they are menstruating.
